# MiR-21-5p but not miR-1-3p expression is modulated by preconditioning in a rat model of myocardial infarction

**DOI:** 10.1007/s11033-020-05721-y

**Published:** 2020-08-12

**Authors:** Annika Raupach, Carolin Torregroza, Julia Niestegge, Katharina Feige, Swantje Klemm-Meyer, Inge Bauer, Timo Brandenburger, Hilbert Grievink, André Heinen, Ragnar Huhn

**Affiliations:** 1 Department of Anesthesiology, University Hospital Duesseldorf, Moorenstr. 5, 40225 Duesseldorf, Germany; 2 Cyclotron/Radiochemistry/MicroPET Unit, Hadassah Hebrew University Hospital, 91120 Jerusalem, Israel; 3 Department of Cardiovascular Physiology, Heinrich-Heine-University Duesseldorf, Universitaetsstr. 1, 40225 Duesseldorf, Germany

**Keywords:** Isoflurane, Preconditioning, miR-1-3p, miR-21-5p, HIF1α

## Abstract

Isoflurane (Iso) preconditioning (PC) is known to be cardioprotective against ischemia/reperfusion (I/R) injury. It was previously shown that microRNA-21-5p (miR-21-5p) is regulated by Iso-PC. It is unclear, if expression of cardiac enriched miR-1-3p is also affected by Iso-PC, and associated with activation of HIF1α (hypoxia-inducible factor 1-alpha).  Male Wistar rats (n = 6–8) were randomly assigned to treatment with or without 1 MAC Iso for 30 min, followed by 25 min of regional myocardial ischemia, with 120 min reperfusion. At the end of reperfusion, myocardial expression of miR-1-3p, miR-21-5p and mRNAs of two HIF-1α-dependent genes, VEGF (vascular endothelial growth factor) and HO-1 (heme oxygenase-1), were determined by quantitative PCR. Protein expression of a miR-21 target gene, PDCD4 (programmed cell death protein 4), was assessed by western blot analysis. Infarct sizes were analyzed with triphenyltetrazoliumchloride staining. MiR-21-5p expression was increased by Iso, whereas expression of miR-1-3p was not altered. The expression of VEGF but not HO-1 was induced by Iso. Iso-PC reduced infarct sizes compared to untreated controls. No regulation of miRNA and mRNA expression was detected after I/R. PDCD4 protein expression was not affected after Iso exposure. Expression of miR-21-5p, in contrast to miR-1-3p, is altered during this early time point of Iso-PC. HIF1α signaling seems to be involved in miR-21-5p regulation.

## Introduction

Cardiac diseases are the leading cause of death in the United States [1] and are initiated or often accompanied by ischemia-reperfusion (I/R) injury. Murry et al. discovered that short intervals of I/R prior to global ischemia reduce I/R injury [2], a phenomenon named ischemic preconditioning (IPC). The effect of IPC can be mimicked pharmacologically. Volatile anesthetics, like isoflurane (Iso), show cardioprotective effects via reducing infarct size in in vivo models [3, 4].

The underlying mechanisms of preconditioning (PC) are yet not fully understood. Evidence suggests, that microRNAs (miRNAs), a class of small non coding RNAs, serve as mediators for preconditioning and influence the protective effect of preconditioning via regulation of apoptosis-related proteins (reviewed in [5]). Yin et al. showed that IPC, followed by I/R, induces expression of miRNAs, e.g. miR-1a-3p, −21a-5p and − 24-3p [6]. In 2015, Qiao et al. demonstrated that isoflurane exposure upregulates miR-21a-5p expression in mouse hearts [7]. Olson et al. underlined this finding with similar results in rat hearts [8]. Mir-21 plays a critical role in Iso-PC demonstrated by a failed reduction in infarct size after Iso-PC in knock out mice of miR-21 [8]. This group also showed that cardioprotection by Iso is mediated by the Akt/nitric oxide synthase (NOS)/mitochondrial permeability transition pore (mPTP) pathway [7]. The expression of miR-21 during PC may be regulated by HIF1α (hypoxia-inducible factor 1-alpha), which was shown through an induction of HIF1α and miR-21 expression induced by hypoxia or renal ischemic preconditioning [9]. Isoflurane is also able to induce HIF1α under normoxic conditions, which was shown in vivo in rat brains and in vitro in primary rat neurons [10]. HIF1α transcriptionally enhances miR-21 promoter activity by binding to its promoter region. Additionally, there exists a regulatory feedback loop via the PTEN (Phosphatase and Tensin homolog)/Akt pathway, which reduces HIF1α expression due to miR-21-5p inhibition [11]. MiR-21-5p itself is able to bind directly to PDCD4 (programmed cell death protein 4), resulting in an anti-apoptotic effect in cardiomyocytes after hypoxia and reoxygenation, mimicking I/R injury in vitro [12]. Zhu et al. supported the assumption that PDCD4 is involved in cardioprotection, showing a clear decrease of PDCD4 protein by ischemic postconditioning in rats [13].

The cardiac enriched miR-1-3p is upregulated during acute myocardial infarction [14] and belongs to the most commonly described regulated miRNAs in the context of preconditioning [5]. Brandenburger et al. showed that miR-1-3p was downregulated by remote IPC alone, I/R, or a combination of both, after 120 min of reperfusion in the area at risk (AAR), while expression remained unaffected in the non-AAR. Interestingly, after 360 min of reperfusion, miR-1-3p expression increased in the non-AAR [15]. This indicates that miR-1-3p expression is time, treatment, and localization dependent.

To our knowledge, it is not clear whether miR-1-3p expression is altered by Iso and if this plays a potential role in the cardioprotective effect of Iso-PC. To analyze these aims we measured miR-1-3p and miR-21-5p expression under (1) isoflurane exposure alone and (2) additional I/R treatment, in rats in vivo. Additionally, (3) possible HIF1α mediated miR-1-3p and miR-21-5p regulation was investigated through two HIF1α targets, VEGF (vascular endothelial growth factor) and HO-1 (heme oxygenase-1), and (4) possible consequences on the direct miR-21-5p target PDCD4 were analyzed.

## Materials and methods

### Animal experiments

In accordance with the German legislation on protection of animals and the National Institutes of Health Guide for the Care and Use of Laboratory Animals (NIH publication 85–23, revised 1996) animal experiments were performed. Experiments were done as described in detail in the study of Heinen et al. [16]. Briefly, 12 weeks old, male Wistar rats were anesthetized by an intraperitoneal pentobarbital (Narcoren, Merial, Germany) injection (80 mg/kg body weight). For maintenance of anesthesia, pentobarbital was infused continuously via a jugular catheter (40 mg/kg/hour). An arterial line was inserted via the left carotid artery. A lateral left-sided thoracotomy was performed, and a suture (5–0) was looped around the left anterior descending coronary artery (LAD).

### Surgery protocol

**Fig. 1:**
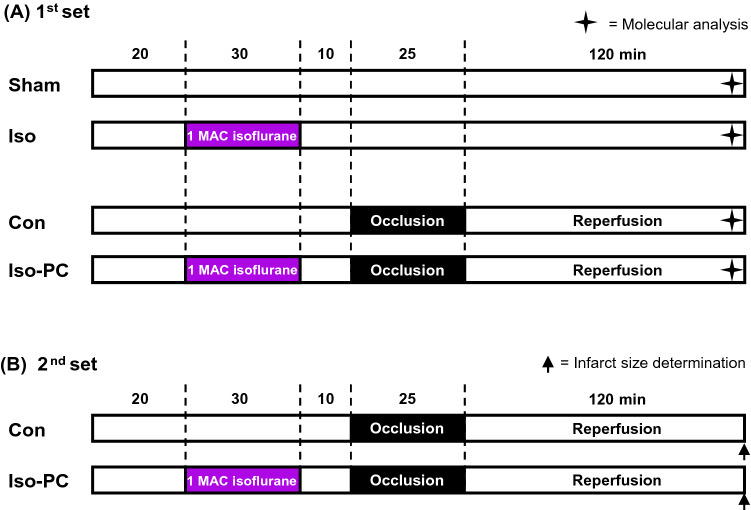
Experimental protocol. **A** 1st set of experiments for harvesting heart tissue for molecular analysis. **B** 2nd set of experiments for infarct size determination. Con control, Iso isoflurane, PC preconditioning

In a first set of experiments, rats were randomized into four groups (n = 6/group; Fig. 1A). The Sham group received anesthesia and thoracotomy, but no further intervention. The Iso group was treated by 30 min inhalation of 1 MAC Iso (1.5% Iso (Baxter Deutschland GmbH, Germany), 40% oxygen in compressed air). Animals in the control group (Con) were not preconditioned before 25 min ischemia of LAD and 120 min reperfusion. Isoflurane preconditioned (Iso-PC) rats received 30 min Iso, with a 10 min washout phase, prior to 25 min ischemia (LAD occlusion) and 120 min reperfusion. At the end of the experiments, each animal was injected with 4 mL Evans blue solution in vivo (after occlusion of the LAD) before heart excision, which allows for the separation of the area at risk (AAR) and the area not at risk (non-AAR). Tissue of the non-AAR was used for further expression analysis and was snap frozen in liquid nitrogen.

For infarct size measurements a second set of experiments was performed (Fig. 1B). Rats were randomized to the protocol of Con (n = 8) and Iso-PC (n = 9). After reperfusion, hearts were perfused with Evans Blue, excised and stained with 0.75% triphenyltetrazoliumchloride (TTC; # 37130.03, Serva, Germany) solution. The infarct size measurement was carried out, using planimetry, by a blinded investigator [17].

During both sets of experiments, hemodynamic parameters were measured continuously, digitized using an analogue to digital converter (PowerLab/8SP, ADInstruments Pty Ltd, Castle Hill, Australia) at a sampling rate of 500 Hz, and recorded on a personal computer using Chart for Windows v5.0 (ADInstruments Pty Ltd, Castle Hill, Australia). Heart rate (bmp) and mean aortic pressure (mmHg) were statistically analyzed during baseline, ischemia, and reperfusion (15, 30, 120 min).

### RNA isolation

From frozen heart tissue total RNA was isolated using TRIzol™ Reagent (Invitrogen™) according to the manufactures instructions. RNA integrity, purity and concentrations were confirmed by agarose gel analysis and spectrophotometry (absorbance at 260 and 280 nm; NanoDrop® ND-1000 (Thermo Scientific, Waltham (Massachusetts, USA)).

### Quantitative polymerase chain reaction (qPCR) assay

Reverse transcription of total RNA was performed using the High Capacity RNA-to-cDNA Master Mix (Applied Biosystems, Life Technologies, Darmstadt, Germany). The qPCR for miRNA expression was performed with TaqMan®MicroRNA Assays 20X (Applied Biosystems, Life Technologies, Darmstadt, Germany), according to the manufacturer’s protocol: rno-miR-1-3p (assay ID: 002064), U6 (assay ID: 001973), and hsa-miR-21-5p (assay ID: 000397). The qPCR for mRNA expression was performed with TaqMan® Gene Expression Assays 20X (Applied Biosystems, Life Technologies, Darmstadt, Germany), according to the manufacturer’s protocol: HO-1 (Rn01536933_m1), VEGF (Rn01511601_m1), GAPDH (Glyceraldehyde 3-phosphate dehydrogenase) (Rn01462661_g1). QPCR conditions were as follows: 50 °C for 2 min, 95 °C for 10 min, 40 cycles of 95 °C for 15 s, 60 °C for 60 s using an ABI 7300HT thermocycler (Applied Biosystems, Life Technologies, Darmstadt, Germany). The relative expression of miRNAs was calculated using the ΔΔCq-method [18].

### Western blot analysis

Frozen heart tissue was homogenized in lysis buffer (20 mM Tris HCl (Sigma-Aldrich, Germany), 150 mM NaCl (Roth, Germany), 1 mM Na-EDTA (Sigma-Aldrich, Germany), 1 mM EGTA (Roth, Germany), 1% NP40, 2.5 mM sodium pyruvate (Sigma-Aldrich, Germany), 2.5 mM sodium vanadate (Sigma-Aldrich, Germany) and freshly added protease inhibitor mix (Complete; Roche, Germany). After determination of protein concentration via the Lowry method [19] the western blot analysis was performed, as previously described in detail [16]. As primary antibodies were used: rabbit anti PDCD4 (#9535, cell signaling, 1:1000) and mouse anti GAPDH (ab8245, abcam, 1:40,000). For detection chemiluminescence was used via the following secondary antibodies purchased from Jackson ImmunoResearch Laboratories Inc.: Peroxidase AffiniPure Donkey Anti-Rabbit IgG (H + L) (#711-035-152, 1:10,000) and Peroxidase AffiniPure Goat Anti-Mouse IgG (H + L) (115-035-003, 1:10,000).

### Statistical analysis

For expression analyses we aimed to detect a minimal difference of 20% by *t*-test. The sample size calculation with a power of 80% (α < 0.05 (two-tailed)), and a within group standard deviation (SD) of 12% predicted a group size of n = 6 [20]. For infarct size analysis we aimed to detect a minimal difference of 25% by *t*-test. The sample size calculation with a power of 80%, α < 0.05, and a within group standard deviation (SD) of 17% predicted a group size of n = 8 [20]. To compare hemodynamic parameters between groups or between different time points within groups, we used a two-way analysis of variance (ANOVA) and a Tukey post hoc test (SPSS Science Software, Version 12.0.1).

Data are presented as mean ± SD. Changes between groups were be considered statistically significant if P < .05.

## Results

### Iso exposure: MiR expression

**Fig. 2:**
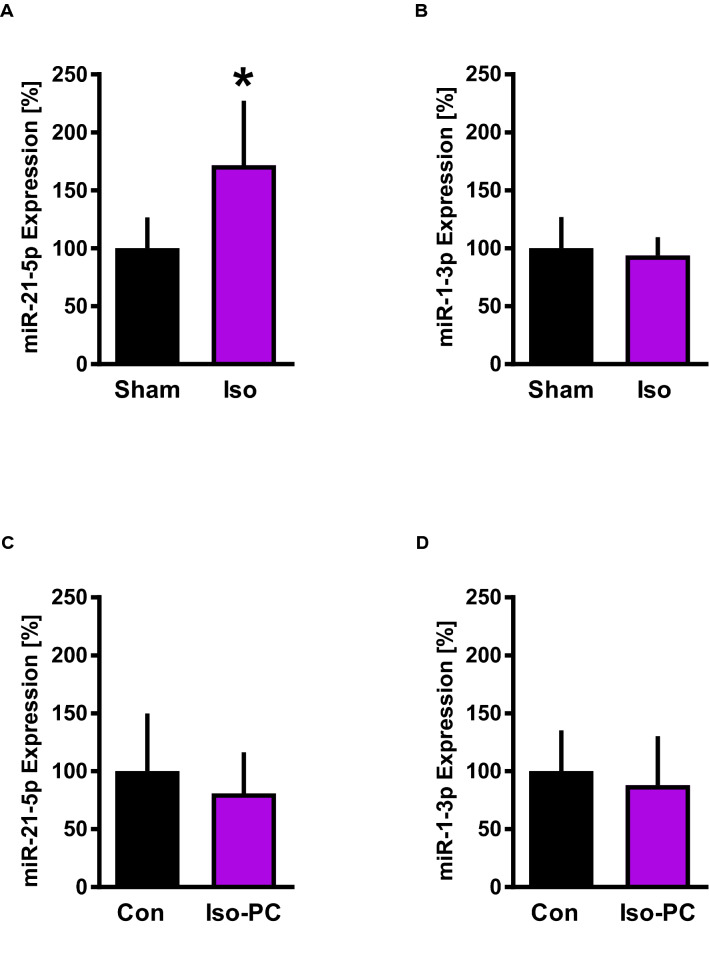
MiR expression analysis in heart tissue. A/B: Expression of miR-21-5p **A** and miR-1-3p **B** without (Sham) or with isoflurane (Iso). C/D: miR-21-5p **C** and miR-1-3p **D** expression following regional myocardial ischemia without (Con) or with (Iso-PC) isoflurane pretreatment. Data are mean ± SD. n = 6 per group. *P < .05 vs Sham

To determine if expression of miR-1-3p is altered by Iso, rats were treated in vivo with or without inhalation of 1 MAC Iso for 30 min and after 155 min heart tissue was analyzed by qPCR. Furthermore, to confirm previous observations using a similar animal model [8], miR-21-5p expression was measured. Iso significantly increased relative miR-21-5p expression compared to Sham (Fig. 2A) (Iso: 172 ± 56% vs. Sham: 100 ± 27%, P < .05). In contrast to miR-21-5p, miR-1 expression levels remained unchanged after Iso exposure (Fig. 2B) (Iso: 94 ± 16% vs. Sham: 100 ± 27%, n.s.). The hemodynamic variables, heart rate and mean aortic pressure, were not different between groups or time points (Table 1).

**Table 1:** Hemodynamic variables (1st set of experiments; molecular analysis)

	Baseline	Ischemia	Reperfusion		
		24	15	60	120
Heart rate (bpm)					
Sham	427 ± 20		409 ± 33	403 ± 20	392 ± 20
Iso	402 ± 20		378 ± 29	390 ± 32	373 ± 18
Con	364 ± 74	407 ± 18	370 ± 94	381 ± 35	342 ± 75
Iso-PC	400 ± 31	392 ± 37	392 ± 39	425 ± 34	384 ± 30
Mean aortic pressure (mmHg)					
Sham	116 ± 7		104 ± 24	102 ± 22	92 ± 30
Iso	118 ± 13		114 ± 17	105 ± 26	81 ± 15
Con	106 ± 17	113 ± 11	117 ± 13	106 ± 5	84 ± 29
Iso-PC	115 ± 37	117 ± 18	116 ± 20	107 ± 24	93 ± 31

Data are mean±SD

*Con*  control,* Iso*  isoflurane,* PC* preconditioning

### Iso preconditioning: MiR expression and infarct sizes

To examine, if Iso followed by I/R influences the expression of miR-21-5p and miR-1-3p in the non-AAR, rats were preconditioned for 30 min with or without isoflurane followed by I/R. The hemodynamic variables were not different between groups or time points (Table 1). Also miR-21-5p and miR-1-3p expression levels were not differently influenced by Iso preconditioning (Fig. 2C and D).

**Fig. 3:**
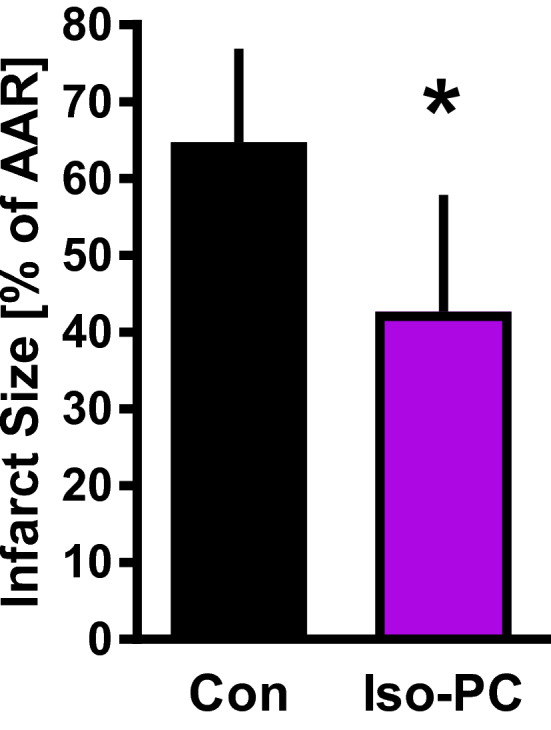
Infarct size measurement. Infarct size of control hearts (Con) and hearts preconditioned with isoflurane (Iso-PC). Data are mean ± SD. Con (n = 8); Iso-PC (n = 9) per group. *P < .05 vs Con

Furthermore, to confirm the cardioprotective effect of Iso-PC in this model, infarct sizes were determined. PC with isoflurane significantly reduced infarct sizes compared to Con (Con: 65 ± 12% vs. Iso-PC: 43 ± 15%, P < .05) (Fig. 3). Hemodynamic variables (Table 2), heart rate and mean aortic pressure, were not different between Con and Iso-PC at baseline or other time point. Mean aortic pressure was only significantly reduced in Iso-PC at 120 min reperfusion compared to baseline.

**Table 2:** Hemodynamic variables (2nd set of experiments; infarct size analysis)

	Baseline	Ischemia	Reperfusion		
		24	15	60	120
Heart Rate (bpm)					
Con	397 ± 24	379 ± 60	378 ± 65	382 ± 43	393 ± 53
Iso-PC	379 ± 41	336 ± 50	340 ± 57	344 ± 57	332 ± 56
Mean aortic pressure (mmHg)					
Con	114 ± 21	116 ± 22	114 ± 29	114 ± 20	102 ± 21
Iso-PC	130 ± 16	102 ± 27	104 ± 28	105 ± 25	86 ± 18^*^

Data are mean±SD

*Con* control,* Iso-PC*  isoflurane preconditioning

*P < .05 vs. Baseline

### HIF1α signaling

**Fig. 4:**
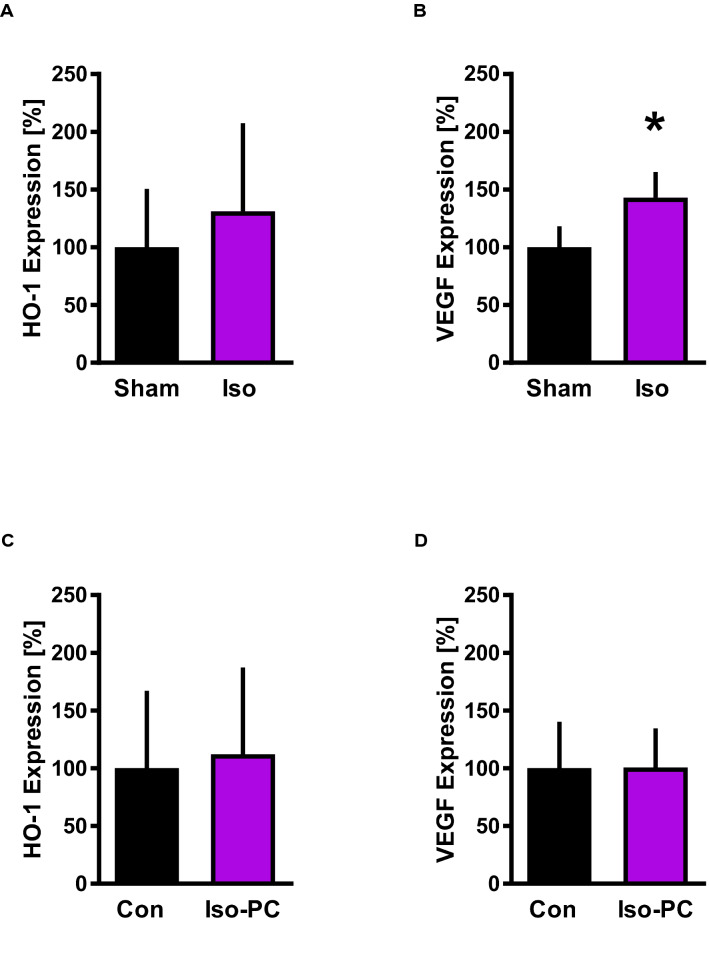
VEGF and HO-1 expression. A/B: HO-1 **A** and VEGF **B** mRNA expression without (Sham) or with isoflurane (Iso). C/D: HO-1 **C** and VEGF **D** mRNA expression following regional myocardial ischemia without (Con) or with isoflurane (Iso-PC). Data are mean ± SD. n = 6 per group. *P < .05 vs Sham

To investigate a potential role of HIF1α in the regulation of miR-1-3p and − 21-5p, HIF1α-activation was analyzed. We used the HIF1α target genes HO-1 and VEGF expression as surrogate parameters for HIF1α-activation [21]. Iso-PC significantly increased VEGF mRNA expression compared to Sham (Iso: 143 ± 22% vs. Sham: 100 ± 18%, P < .05; Fig. 4B), while HO-1 mRNA levels remained unaltered (Iso: 131 ± 76% vs. Sham: 100 ± 51%; Fig. 4A). Additionally, subsequent I/R intervention revealed similar mRNA expression levels of HO-1 (Iso-PC: 112 ± 75% vs. Con: 100 ± 67%, n.s.) and VEGF (Iso-PC: 101 ± 34% vs. Con: 100 ± 40%, n.s.), with or without Iso exposure (Fig. 4C and D).

#### PDCD4

**Fig. 5:**
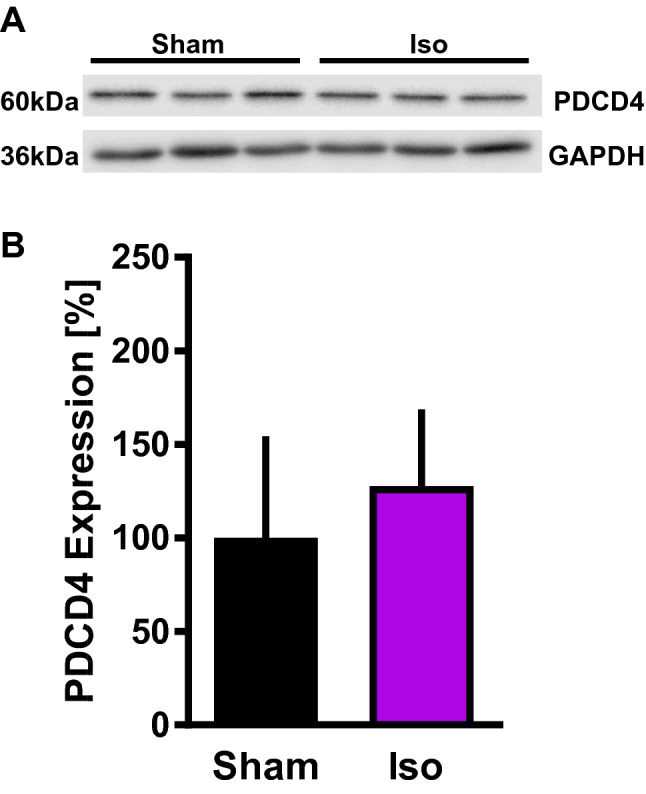
PDCD4 protein expression without (Sham) or with isoflurane (Iso) pretreatment. **A** Representative western blot analysis. GAPDH served as loading control. **B** Quantification of western blot analysis. Data are mean ± SD. n = 6 per group

As a direct target of miR-21-5p we analyzed the protein expression of PDCD4 [9]. Protein amounts of PDCD4 were not affected by Iso treatment compared to Sham (Iso: 128 ± 41% vs. Sham: 100 ± 54%; n.s.; Fig. 5).

## Discussion

The results of the present study demonstrate increased miR-21-5p expression in rat hearts by isoflurane. In contrast, the expression of miR-1-3p was not changed. The expression of HIF1α target VEGF, but not HO-1, was induced by isoflurane. No differences were detected after I/R. The miR-21-5p target PDCD4 was not affected by the increase of miR-21-5p after isoflurane exposure.

Olson et al. showed an increase of miR-21-5p expression shortly (15 min) after exposure to 30 min Iso [8], whereas in this study the expression was measured 155 min after Iso exposure. Interestingly, this increase was not detectable after I/R in the non-AAR at the same time point. I/R seems to abrogate the miR-21-5p increase in the non-AAR. However, the literature on miR-21-5p is inconsistent and differs depending on the experimental setup and statistical comparisons. Rooji et al. found an upregulation of miR-21-5p three and fourteen days after acute myocardial injury in the border zone and remote myocardium, in comparison to sham operated mice, indicating an upregulation through I/R injury itself [22]. Dong et al. showed a downregulation of miR-21-5p in infarcted areas, but an increase in the border zone, compared to non-infarcted areas [23]. A potential effect of Iso, on miR-21-5p expression after I/R, could have been masked by a concomitant increase of miR-21-5p expression due to I/R itself. This could explain the lack of difference in miR-21-5p in Iso-PC vs. Con. In this study, we focused on the comparison of groups with and without Iso. Interestingly, IPC itself increased miR-21-5p expression and in infarcted hearts IPC inhibited downregulation of miR-21-5p after 6 h of acute myocardial infarction compared to sham control [23]. Taken together, miR-21-5p expression seems to be dependent on the preconditioning mode, measurement time point and tissue area.

The increase of VEGF expression, as an indirect marker of HIF1α activity, by Iso exposure indicates that HIF1α could be responsible for miR-21-5p upregulation. This is in line with the findings of Wang et al. showing that protein expression of VEGF, HIF1α and extracellular signal-regulated kinase (Erk) increased shortly (15 min) after Iso exposure and was sustained at least up to 155 min after Iso exposure [24]. Additionally, Jiang et al. also demonstrated that Iso induces HIF1α expression [10]. These findings suggest that HIF1α is involved in Iso-PC.

An increase of miR-1-3p expression due to Iso exposure was not detected, indicating that miR-1-3p does not seem to play a critical role in Iso-PC. But there is evidence for a role of miR-1-3p in other forms of PC. Brandenburger et al. showed that remote IPC (RIPC) alone led to an early downregulation (165 min after conditioning), but later upregulation (405 min) of miR-1-3p expression [15]. Additional I/R intervention did not influence miR-1-3p expression at the early time point. In contrast, after 6 h miR-1-3p expression was upregulated indicating a time-dependent regulation of miR-1-3p in the non-AAR after I/R. Dong et al. also found an upregulation of miR-1-3p levels after 6 h, in an *in vivo* rat model of left coronary artery ligation [23]. In this study, miR-1-3p expression was measured after 2.5 h Iso exposure which was possibly too early to detect an influence on expression. Thus, at early time points miR-1-3p expression does not seem to be regulated by Iso, but a regulation at later time points might be possible.

To examine a downstream response of miR-21-5p upregulation, the protein expression of PDCD4 was analyzed. It was previously shown that knockdown of miR-21 increased PDCD4 expression, and the activity of its own downstream target NF-κB (nuclear factor-kappa B), in mice organs (e.g. heart and kidney) [9]. In this study, upregulation of miR-21-5p was not associated with a decrease in the protein expression of PDCD4. This could be due to the time point of tissue harvesting. The time span is possibly too short to detect changes in protein expression and other studies used later time points. Two independent groups measured the PDCD4 expression 24 h after knockdown of miR-21 expression in mice organs [9] or isolated rat cardiomyocytes [23] and found an upregulation of PDCD4 expression. The latter group could also show that PDCD4 has a pro-apoptotic effect, which was suppressed by miR-21-5p resulting in the miR-21-5p cardioprotective effect during myocardial infarction [23]. PDCD4 expression is not only regulated by miR-21-5p, but also at the level of transcription, translation and protein degradation [25], therefore mechanisms influenced by isoflurane with opposing effects than miR-21-5p could be responsible for maintaining the PCDC4 expression. For example, isoflurane is able to increase transforming growth factor beta-1 (TGF-β1) levels [26], which in turn induces apoptosis via PDCD4 overexpression [25].

## Conclusions

MiR-1-3p does not seem to play a critical role during the early phase of Iso-PC. In this period miR-21-5p signaling appears to be more prominent.

## Data Availability

The datasets used and/or analyzed during the current study are available from the corresponding author on reasonable request.

## References

[1] Virani SS, Alonso A, Benjamin EJ, Bittencourt MS, Callaway CW, Carson AP, Chamberlain AM, Chang AR, Cheng S, Delling FN, Djousse L, Elkind MSV, Ferguson JF, Fornage M, Khan SS, Kissela BM, Knutson KL, Kwan TW, Lackland DT, Lewis TT, Lichtman JH, Longenecker CT, Loop MS, Lutsey PL, Martin SS, Matsushita K, Moran AE, Mussolino ME, Perak AM, Rosamond WD, Roth GA, Sampson UKA, Satou GM, Schroeder EB, Shah SH, Shay CM, Spartano NL, Stokes A, Tirschwell DL, VanWagner LB, Tsao CW, Stroke Statistics C, American Heart Association Council on E, Prevention Statistics (2020). Heart Disease and Stroke Statistics-2020 Update: A Report From the American Heart Association. Circulation.

[2] Murry CE, Jennings RB, Reimer KA (1986). Preconditioning with ischemia: a delay of lethal cell injury in ischemic myocardium. Circulation.

[3] Tonkovic-Capin M, Gross GJ, Bosnjak ZJ, Tweddell JS, Fitzpatrick CM, Baker JE (2002). Delayed cardioprotection by isoflurane: role of K(ATP) channels. Am J Physiol Heart Circ Physiol.

[4] Kersten JR, Schmeling TJ, Pagel PS, Gross GJ, Warltier DC (1997). Isoflurane mimics ischemic preconditioning via activation of K(ATP) channels: reduction of myocardial infarct size with an acute memory phase. Anesthesiology.

[5] Kohns M, Huhn R, Bauer I, Brandenburger T (2018). Mirna-Mediated Mechanisms of Cardiac Protection in Ischemic and Remote Ischemic Preconditioning - A Qualitative Systematic Review. Shock.

[6] Yin C, Salloum FN, Kukreja RC (2009). A novel role of microRNA in late preconditioning: upregulation of endothelial nitric oxide synthase and heat shock protein 70. Circ Res.

[7] Qiao S, Olson JM, Paterson M, Yan Y, Zaja I, Liu Y, Riess ML, Kersten JR, Liang M, Warltier DC, Bosnjak ZJ, Ge ZD (2015). MicroRNA-21 Mediates Isoflurane-induced Cardioprotection against Ischemia-Reperfusion Injury via Akt/Nitric Oxide Synthase/Mitochondrial Permeability Transition Pore Pathway. Anesthesiology.

[8] Olson JM, Yan Y, Bai X, Ge ZD, Liang M, Kriegel AJ, Twaroski DM, Bosnjak ZJ (2015). Up-regulation of microRNA-21 mediates isoflurane-induced protection of cardiomyocytes. Anesthesiology.

[9] Jia P, Wu X, Dai Y, Teng J, Fang Y, Hu J, Zou J, Liang M, Ding X (2017). MicroRNA-21 Is Required for Local and Remote Ischemic Preconditioning in Multiple Organ Protection Against Sepsis. Crit Care Med.

[10] Jiang H, Huang Y, Xu H, Sun Y, Han N, Li QF (2012). Hypoxia inducible factor-1alpha is involved in the neurodegeneration induced by isoflurane in the brain of neonatal rats. J Neurochem.

[11] Liu Y, Nie H, Zhang K, Ma D, Yang G, Zheng Z, Liu K, Yu B, Zhai C, Yang S (2014). A feedback regulatory loop between HIF-1alpha and miR-21 in response to hypoxia in cardiomyocytes. FEBS Lett.

[12] Cheng Y, Zhu P, Yang J, Liu X, Dong S, Wang X, Chun B, Zhuang J, Zhang C (2010). Ischaemic preconditioning-regulated miR-21 protects heart against ischaemia/reperfusion injury via anti-apoptosis through its target PDCD4. Cardiovasc Res.

[13] Zhu J, Yao K, Wang Q, Guo J, Shi H, Ma L, Liu H, Gao W, Zou Y, Ge J (2016). Ischemic Postconditioning-Regulated miR-499 Protects the Rat Heart Against Ischemia/Reperfusion Injury by Inhibiting Apoptosis through PDCD4. Cell Physiol Biochem.

[14] Ai J, Zhang R, Li Y, Pu J, Lu Y, Jiao J, Li K, Yu B, Li Z, Wang R, Wang L, Li Q, Wang N, Shan H, Li Z, Yang B (2010). Circulating microRNA-1 as a potential novel biomarker for acute myocardial infarction. Biochem Biophys Res Commun.

[15] Brandenburger T, Grievink H, Heinen N, Barthel F, Huhn R, Stachuletz F, Kohns M, Pannen B, Bauer I (2014). Effects of remote ischemic preconditioning and myocardial ischemia on microRNA-1 expression in the rat heart in vivo. Shock.

[16] Heinen NM, Putz VE, Gorgens JI, Huhn R, Gruber Y, Barthuber C, Preckel B, Pannen BH, Bauer I (2011). Cardioprotection by remote ischemic preconditioning exhibits a signaling pattern different from local ischemic preconditioning. Shock.

[17] Behmenburg F, Dorsch M, Huhn R, Mally D, Heinen A, Hollmann MW, Berger MM (2015). Impact of Mitochondrial Ca2+-Sensitive Potassium (mBKCa) Channels in Sildenafil-Induced Cardioprotection in Rats. PLoS One.

[18] Pfaffl MW (2001). A new mathematical model for relative quantification in real-time RT-PCR. Nucleic Acids Res.

[19] Lowry OH, Rosebrough NJ, Farr AL, Randall RJ (1951). Protein measurement with the Folin phenol reagent. J Biol Chem.

[20] Chow SSJ, Wang H (2008) Sample Size Calculations in Clinical Research. 2nd Ed.Chapman & Hall/CRC Biostatistics Series. page 58

[21] Berger MM, Huhn R, Oei GT, Heinen A, Winzer A, Bauer I, Preckel B, Weber NC, Schlack W, Hollmann MW (2010). Hypoxia induces late preconditioning in the rat heart in vivo. Anesthesiology.

[22] van Rooij E, Sutherland LB, Thatcher JE, DiMaio JM, Naseem RH, Marshall WS, Hill JA, Olson EN (2008). Dysregulation of microRNAs after myocardial infarction reveals a role of miR-29 in cardiac fibrosis. Proc Natl Acad Sci U S A.

[23] Dong S, Cheng Y, Yang J, Li J, Liu X, Wang X, Wang D, Krall TJ, Delphin ES, Zhang C (2009). MicroRNA expression signature and the role of microRNA-21 in the early phase of acute myocardial infarction. J Biol Chem.

[24] Wang C, Weihrauch D, Schwabe DA, Bienengraeber M, Warltier DC, Kersten JR, Pratt PF, Pagel PS (2006). Extracellular signal-regulated kinases trigger isoflurane preconditioning concomitant with upregulation of hypoxia-inducible factor-1alpha and vascular endothelial growth factor expression in rats. Anesth Analg.

[25] Matsuhashi S, Manirujjaman M, Hamajima H, Ozaki I (2019). Control Mechanisms of the Tumor Suppressor PDCD4: Expression and Functions. Int J Mol Sci.

[26] Yin J, Liu X, Wang R, Ge M, Xie L, Zhai J, Dai Z, Li Y, Wang S (2020). Isoflurane postconditioning upregulates phosphorylated connexin 43 in the Middle Cerebral Artery occlusion model and is probably associated with the TGF-beta1/Smad2/3 signaling pathway. Biomed Res Int.

